# Supplementary material for: Drivers of Diagnostic Delay in Mitochondrial Disease: Missed Recognition of Canonical Features
Source: medRxiv. 2025 Oct 14:2025.10.09.25337582. Preprint. [Version 1] doi: 10.1101/2025.10.09.25337582 (PMC12633123; doi:10.1101/2025.10.09.25337582)
Supplement: 1 [file NIHPP2025.10.09.25337582V1-supplement-1.pdf]

medRxiv preprint doi: https://doi.org/10.1101/2025.10.09.25337582; this version posted October 14, 2025. The copyright holder for this preprint (which was not certified by peer review) is the author/funder, who has granted medRxiv a license to display the preprint in perpetuity.
It is made available under a CC-BY 4.0 International license .

Supplementary Figures and table Legends

Supplementary Figure 1a–b.
Expanded visualizations of additional clinical features that were not significantly associated with diagnostic delay. Binary features are displayed as violin plots comparing individuals without (0) and with (1) the feature; the red diamond marks the

medRxiv preprint doi: https://doi.org/10.1101/2025.10.09.25337582; this version posted October 14, 2025. The copyright holder for this preprint (which was not certified by peer review) is the author/funder, who has granted medRxiv a license to display the preprint in perpetuity.
It is made available under a CC-BY 4.0 International license .

group mean, and boxplots indicate the median and interquartile range. Heteroplasmy (%) is shown as a scatterplot with fitted regression line and 95% confidence band. None of the associations reached statistical significance ($p < 0.05$).

Supplementary Tables

Supplementary Table 1. Predictors of diagnostic delay in mitochondrial disease. Summary of demographic, genetic, and clinical features tested for association with diagnostic delay. The table reports sample size, test statistics, and p-values for each factor. Significant predictors included developmental delay (associated with longer diagnostic delay) and family history of similar symptoms (associated with shorter diagnostic delay). Other variables, including sex, nuclear versus mitochondrial genetic etiology, seizures, cardiomyopathy, muscle weakness, and stroke-like episodes, did not reach statistical significance.

Supplementary Table 2
Family Demographics of individuals who were asymptomatic but were tested because of family history. The proband was the individual who presented with symptoms and initiated cascade testing. The parent of Family 3 had an affected sibling who was not seen in our clinic. DM2=Diabetes Mellitus Type II. GDD=Global Developmental Delay, SNHL=Sensorineural Hearing Loss, N/A=Asymptomatic Individuals.

Supplementary Table 3. Frequency and lead time of HPO terms documented prior to clinical suspicion

Summary of all HPO terms identified in the electronic health record (EHR) with documentation before and after the point of clinical suspicion for mitochondrial disease. For each feature, the table provides the proportion of patients with documentation before and after suspicion, the number of patients contributing lead time data, and the mean and median years the feature was recorded prior to diagnosis. Inclusion in the analysis required that each individual have at least one HPO term documented before suspicion, to account for data fragmentation across records.

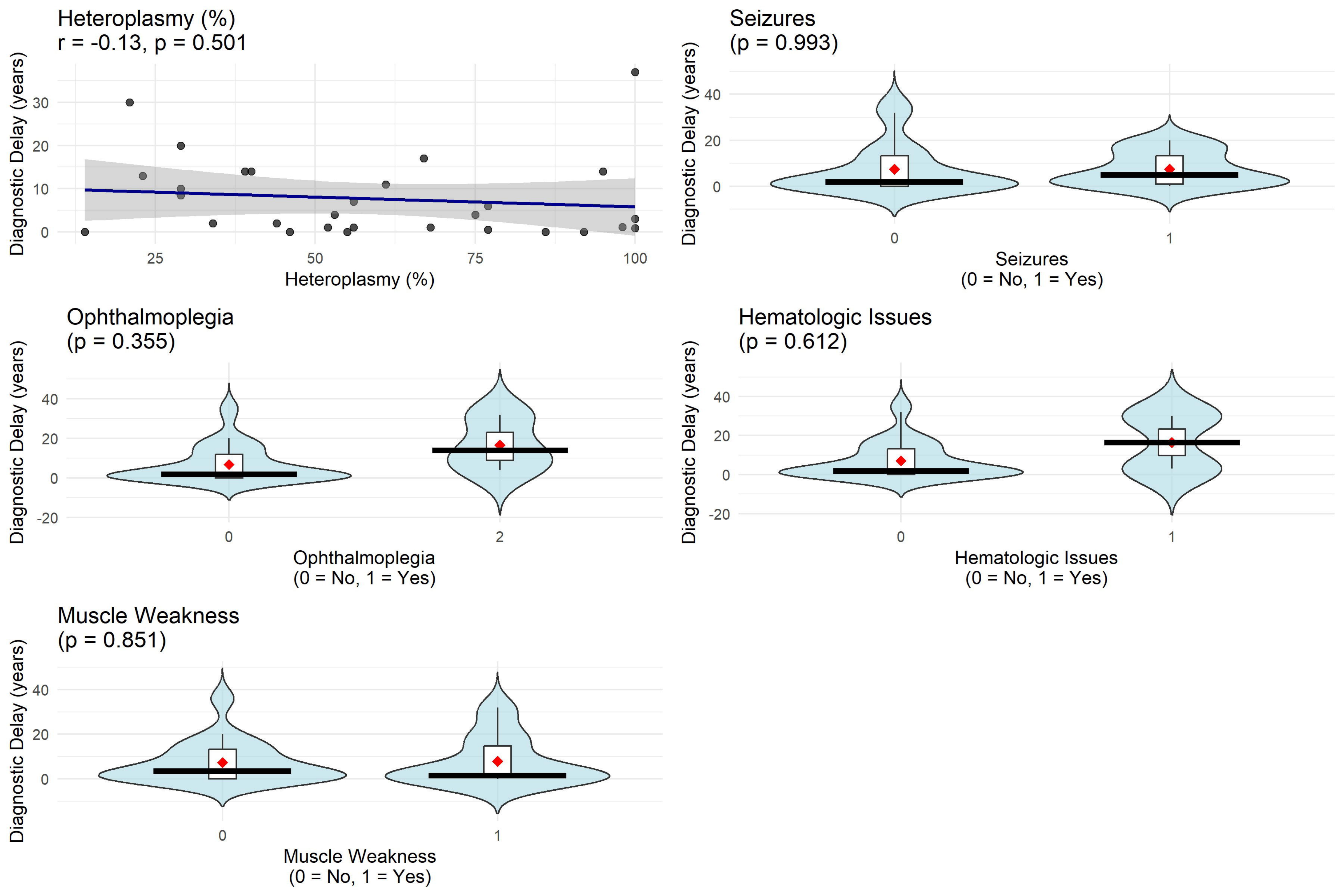
Heteroplasmy (%)
r = -0.13, p = 0.501
Diagnostic Delay (years)
Heteroplasmy (%)
Seizures
(p = 0.993)
Seizures
(0 = No, 1 = Yes)
Ophthalmoplegia
(p = 0.355)
Ophthalmoplegia
(0 = No, 1 = Yes)
Hematologic Issues
(p = 0.612)
Hematologic Issues
(0 = No, 1 = Yes)
Muscle Weakness
(p = 0.851)
Muscle Weakness
(0 = No, 1 = Yes)

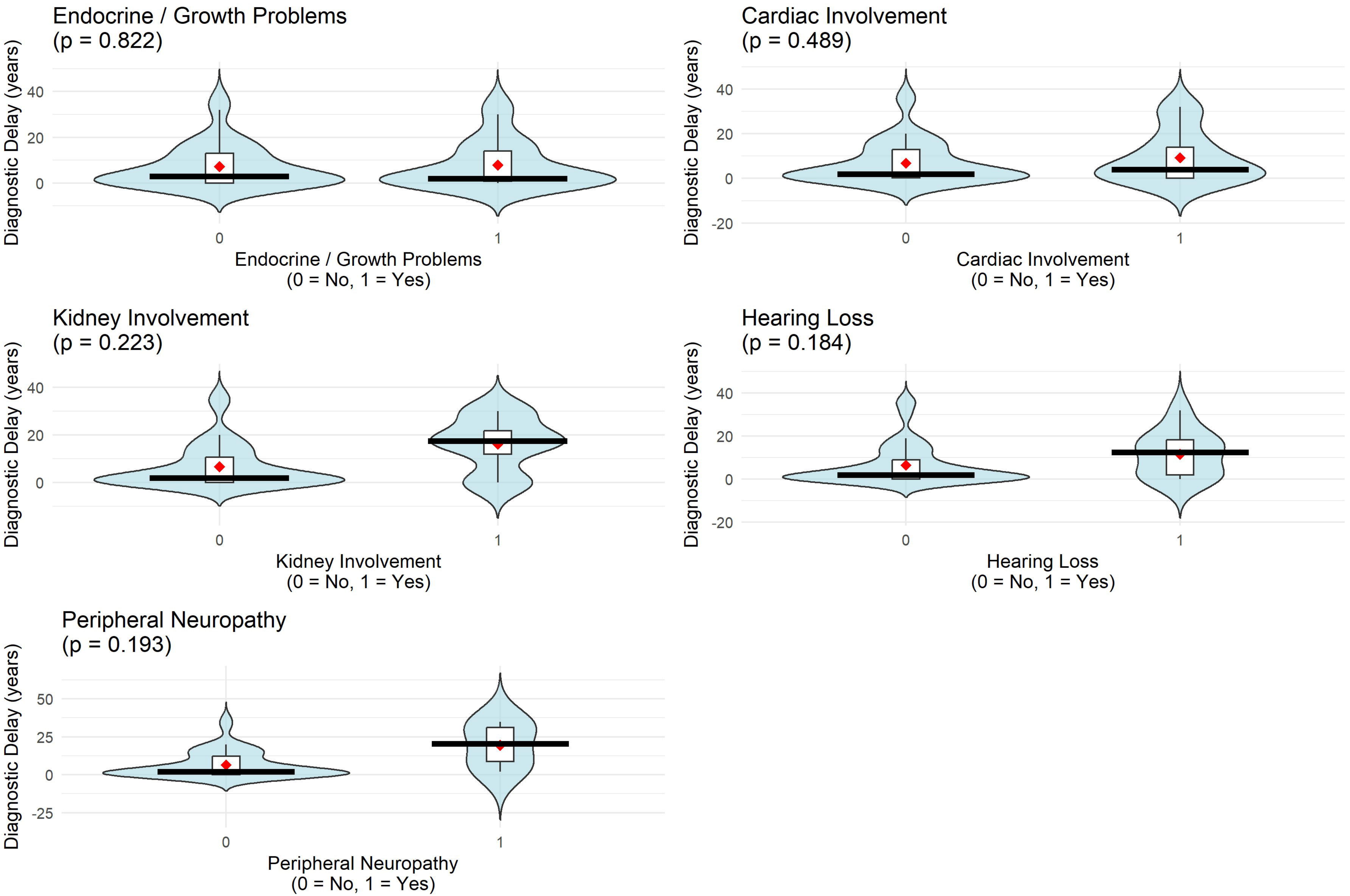

Endocrine / Growth Problems
(p = 0.822)
Diagnostic Delay (years)
40
20
0
0
1
Endocrine / Growth Problems
(0 = No, 1 = Yes)
Cardiac Involvement
(p = 0.489)
Diagnostic Delay (years)
40
20
0
-20
0
1
Cardiac Involvement
(0 = No, 1 = Yes)
Kidney Involvement
(p = 0.223)
Diagnostic Delay (years)
40
20
0
0
1
Kidney Involvement
(0 = No, 1 = Yes)
Hearing Loss
(p = 0.184)
Diagnostic Delay (years)
40
20
0
-20
0
1
Hearing Loss
(0 = No, 1 = Yes)
Peripheral Neuropathy
(p = 0.193)
Diagnostic Delay (years)
50
25
0
-25
0
1
Peripheral Neuropathy
(0 = No, 1 = Yes)
